# The effects of dietary diversity on health status among the older adults: an empirical study from China

**DOI:** 10.1186/s12889-024-18172-y

**Published:** 2024-03-03

**Authors:** Yali Zhu, Qiaozhen An, Jiahao Rao

**Affiliations:** School of Economics and Management, China University of Geosciences, No.388 Lumo Road, Hongshan District, 430000 Wuhan, Hubei Province China

**Keywords:** Dietary diversity, Health status, Older adults, Lifestyle, Multinomial logistic regression

## Abstract

**Background:**

Dietary diversity is an indicator of nutrient intake among the elderly. Previous researches have primarily examined dietary diversity and the risks with chronic and infectious disease and cognitive impairment, limited evidence shows the association between dietary diversity and the overall health status of specific populations with a heterogeneity analysis. This study aimed to probe the effects of dietary diversity on health status among Chinese older adults.

**Methods:**

There were 5740 sample participants aged 65 and above selected from the Chinese Longitudinal Healthy Longevity Survey, among which 3334 samples in 2018 wave and 2406 samples in 2011 wave. Dietary diversity was assessed by Dietary Diversity Score ranged from 0 to 9, the higher the score, the better dietary diversity. Health status was assessed into healthy, impaired and dysfunctional state by three indicators: Activities of Daily Living, Instrument Activities of Daily Living and Mini-Mental State Examination. Multinomial logistic regression was employed to assess the effects of dietary diversity on the health status among the elderly. Heterogeneity analysis between different groups by age was further discussed.

**Results:**

Older adults with better dietary diversity are in better health status, the mean dietary diversity score for healthy group was higher than that of impaired and dysfunctional groups (In 2018 wave, the scores were 6.54, 6.26 and 5.92, respectively; and in 2011 wave, they were 6.38, 5.93 and 5.71, respectively). Heterogeneity analysis shows that the younger groups tend to have more diversified dietary and be in better health status. Dietary diversity was more significantly associated with health status of the younger elderly (OR, 1.22, 95% CI, 1.04–1.44, *p* < 0.05) than the older elderly (OR, 1.01, 95% CI, 0.37–2.78, *p* > 0.05) in 2018 wave; and in 2011 wave, dietary diversity was more significantly related to health status among the younger elderly (OR, 1.62, 95% CI, 1.26–2.08, *p* < 0.001) than the older elderly (OR, 0.08, 95%CI, 0.31–1.94, *p* > 0.05).

**Conclusions:**

Better dietary diversity has positive effects on health status and is more significantly related to the younger elderly than the older elderly. So interventions including available dietary diversity assessment, variety of dietary assistance services in daily life, keeping nutrient digestion and absorption capacity for the venerable age might benefit to ensure the effects of dietary diversity on health status among older adults especially in maintaining intrinsic ability and physical function. In addition, healthy lifestyle should also be recommended.

## Introduction

The 21st century is an irreversible aging society globally, virtually every country in the world is experiencing growth in the size and proportion of the older adults in their population. The aging population exceeded 1.04 billion by 2020 and it will reach 2.08 billion by 2050 accounting for 21.3% of the total population globally according to the United Nations projection. Health status of the older adults is becoming a key concern as population aging accelerates worldwide [1]. Nutrition is essential to human being, with the growth of age, the elderly will have progressive and systemic changes in the body, which will be reflected in the digestive system, nervous system and other systems of the body, resulting in the elderly facing quite severe nutritional and health conditions [2].

As one of the most populous countries, China is experiencing an unprecedented population aging, at the end of 2023, population aged 60 and above in China reached 296.97 million accounting for 21.1% of the total population [3]. However, “China Older Adults Nutrition and Health Report” shows that 48.4% of the elderly are in poor nutritional status. Researchers have found out that diversified diet is inversely associated with all-cause mortality among the elderly in China, especially the oldest old and men [4], and poor food diversity is associated with poorer cognitive function in Chinese elderly [5].

Nutritionists point out that by increasing dietary diversity (DD), it helps to increase the diversity of intestinal microbiota, ensure adequate nutrient intake, improve dietary quality, and thus improve the nutritional status of the body [6, 7]. Better DD means more comprehensive and adequate nutrient intake and better nutritional conditions [8], which helps to maintain physical function and has a protective effect against the decline in multidimensional outcomes with healthy aging. Diversified diets are positively related to better cognitive function, slower cognitive decline and reduced risk of dementia [9], and can reduce the risk of chronic diseases (cardiovascular and cerebrovascular diseases, etc.) [10]. However, poor DD may lead to adverse effects such as insufficient nutrient intake and reduced energy storage, thus results in impaired immune function and increased infectious and chronic diseases [11]. In addition, lack of DD is a particularly severe problem in developing countries where diets are predominantly based on starchy staples and little or no animal products. These plant-based diets tend to be low in a number of micronutrients [12].

In practice, daily intake of a variety of foods is beneficial to preventing declines in cognitive ability and intellectual activity among the elderly in the community [13–15]. For example, adhering to the Mediterranean-DASH intervention for Neurodegenerative Delay (MIND) diet may be associated with improved cognitive function in older adults [16].

Previous research mainly focused on the relationship between DD and chronic and infectious diseases, or cognitive impairment alone and separately among the older adults [17, 18], little was done on the effects of DD on health status among the older adults comprehensively from both physical and cognitive aspects simultaneously to afford implications to behavioral interventions in practice. In fact, focusing on health rather than disease is more beneficial to the elderly, because the best way to prevent disease is by promoting health, and behavioral intervention may be a low-cost way to improve health status among the elderly, especially the maintenance of functional ability throughout the lifespan.

This study was designed to explore the effects of DD on health status among the elderly, taking China as an example. Firstly, the effects of DD on health status of the elderly was analyzed by using multinomial logistic regression together with data of CLHLS 2018 and 2011 wave. And then the effect of lifestyles includes smoking, drinking and physical exercise on health status was also explored. Further, heterogeneity analysis between different age groups was discussed. These results can provide a practice reference in guiding behaviour intervention to promote health among the elderly as population aging accelerates.

## Methods

### Design and participants

Data of CLHLS used in this paper is the survey result organized by Peking University. The questionnaire survey adopted the multi-stage cluster sampling method, it covers comprehensive information of older adults from 23 provinces in China, including demographic information, health status, food and nutrition intake, lifestyles, family and social data of the elderly with written informed consents from all participants. And the data has been proved of high quality [19, 20]. The baseline survey of CLHLS data began in 1998, followed by micro tracking surveys conducted in 2000, 2002, 2005, 2008–2009, 2011–2012, 2014, and 2017–2018. Dietary Guidelines was firstly issued in China in 1989 and revised in 1997, 2007, 2016 and 2022. Taking into account the changes in dietary structure in China and the guidance on dietary diversity by Chinese Nutrition Society, this study chose the CLHLS data of 2011 and 2018 wave in the empirical analysis.

Considering that the age structure of the elderly in CLHLS 2011and 2018 wave may not necessary consistent with the actual situation, directly use of this sample data in the following empirical analysis may result in biased result due to age structure deviation, so we reconstructed the sample data of CLHLS 2011and 2018 wave according to the age and gender distribution of data from World Population Prospects 2022 (WPP 2022) released by the United Nations,.so that the distribution of age and gender of aged population in the reconstructed sample could be more consistent with the actual distribution. In order to avoid biased estimation caused by a particular sampling, reconstruction of the samples was performed 2,000 times.

The elderly aged 65 and above were the target population, and from CLHLS data, 2406 qualified samples of 2011 wave and 3334 qualified samples of 2018 wave were selected after sampling and processing the missing data by multiple imputations, while samples were excluded due to missing of key variables, such as health status and diet information.

### Assessment of health status

The response variable was the health status of interviewed older adults, referring to Cheng et al. (2015) [21], three indicators were used to measure health status among the older adults synthetically, namely Activities of Daily Living (ADL) [22], Instrument Activities of Daily Living (IADL) [23] and Mini-Mental State Examination (MMSE) [24].

ADL and IADL were used to assess physical health, the more items listed in ADL and IADL could be accomplished independently by older adults without difficulties, the better physical health status. ADL mainly contains six items, including bathing, dressing, activities, going to the toilet, eating, urine and stool control. IADL contains 8 items, including going out for activities, shopping, independent cooking, independent laundry, continuous walking, continuous lifting of heavy objects, continuous squatting, independent riding. MMSE was used to assess mental or cognitive health of the older adults. There are 24 questions in MMSE scoring 30 points in total, the higher the score, the higher level of the mental health or cognitive function. Scoring 24 points and above indicates a good level, scoring 21–23 points indicates a medium level and scoring less than 21 points indicates a poor level as adopting the standards by Shahid et al. (2011) [25].

Health status of living older adults was assessed and classified into three states. If all items listed in ADL and IADL could be accomplished independently by the older adults, it defined as a healthy state; otherwise, if 1 or 2 items could not be accomplished independently, it defined as an impaired state; 3 or more items could not be accomplished independently or MMSE scoring below 21 points, it defined as a dysfunctional state.

### Assessment of dietary diversity

Dietary Diversity (DD) was the explanatory variable. Dietary Diversity Score (DDS) is often used as an evaluation index of dietary diversity [26–29], which is especially suitable for rural population and elderly population, and it can also be seen as a propaganda tool to pay more attention to vulnerable groups [30].

Referring to the Chinese Dietary Guidelines (2022), this paper classified all foods into 9 main kinds: cereal, vegetables, fruits, soybeans and its products, eggs, meat, fish, milk and dairy products and oil. From dietary data in CLHLS, all these 9 kinds could be chosen for the Dietary Diversity Score-9 (DDS-9) assessment. The food intake frequency was sorted into five grades based on the interviewee’s answers: “almost every day”, “not every day, but once a week at least”, “not every week, but once a month at least”, “not every month, but sometimes”, “seldom or never”. If the answer was “almost every day” or “once a week at least”, it scored 1 point, otherwise scored 0 point. The same main kind of foods only scored once, and the higher score of DDS, the better level of DD, the highest score was 9 points.

### Covariates

Health can be affected by many factors as proved by the theory of health production [31], so the covariates of demographic characteristics (age, gender, education years, marital status, income and residence) and lifestyles (smoking, drinking and physical exercise) were collected from data of CLHLS. Education years, as an indicator of socioeconomic status [32] was classified into 0 year (no formal education), 1–6 years (primary school) and more than 6 years (middle school or higher). Marital status was divided into two states, namely married and not married (divorced, widowed and never married included). Physical exercise was assessed by the interviewee’s answers to two questions: (1) “Do you exercise regularly (that are purposeful fitness activities, such as walking, playing balls, running, Qigong and so on)?” The answers included “yes” or “no”. (2) “Do you take outdoor activities?” The five-point scale was used to categorize its answers by “almost every day”, “once a week at least”, “once a month at least”, “sometimes”, “seldom or never”. If the answer was “almost every day” or “once a week at least”, it categorized as “yes”, otherwise as “no”. Physical exercise was defined as “yes” if the answer to one of the two questions included “yes”.

### Statistical analysis

Analysis of Variance (ANOVA) for continuous variables and chi-square test for categorical variables were used to compare characteristics of the older adults with different health states. The DDS distribution of the samples were normal because there was no established cut-off points in terms of number of food groups to indicate DD [5], so the study population were divided into two groups as better DD and poor DD with the mean of DDS among samples based on suggestions of FAO (Food and Agriculture Organization), for those higher than mean were classified as “better DD”, otherwise defined as “poor DD”.

Multinomial logistic regression is suitable for discussing the relationship of multiple categorical variables, so it was used to analyze the effects of DD on health status. There were a set of models in this study, model 1 was adjusted by demographic variables (age, gender, educational years, marital status, income); model 2 was further adjusted by smoking, drinking and physical exercise. Afterwards, in order to test the robustness of the results, target population were categorized into three groups by DDS: Scoring 0–3 points was classified as insufficient DD group, scoring 4–6 points as moderate DD group, and scoring 7–9 points as sufficient DD group, and then made the same statistical analysis. All analyses were performed by using Stata 17.0 and SPSS 22.0, values of *p* < 0.05 were considered as statistically significant and all reported p values were two-sided.

## Results

The target population with different health states is showed in Table 1. There were 3334 samples in 2018 wave, among which 1519 samples were male, and the number in healthy, impaired and dysfunctional health states was 1906, 713 and 715, respectively. The mean of DDS was 6.35 and there were 1635 older adults in better DD group. For the sample situation of 2011 wave, this paper didn’t list in detail, only the statistical significance results of relevant variables were listed in Table 1.

Older adults in impaired and dysfunctional groups were elder in age and less educated than that in the healthy group, and they tended to smoke and drink less at present; and the size of married older adults in impaired and dysfunctional groups was smaller than that in healthy group. The mean of DDS in healthy group was higher than that in impaired and dysfunctional groups (The mean of DDS in healthy, impaired and dysfunctional groups in 2018 wave were 6.54, 6.26 and 5.92, respectively; and in 2011 wave, they were 6.38, 5.93 and 5.71, respectively). Except residence factor, other explanatory variables and concomitant variables were all statistically significant (*p* < 0.01) (Table 1).

**Table 1:** Descriptive statistics of health status

Characteristics	Health status (2018)					2011
	Total sample	Healthy	Impaired	Dysfunctional	P value	P value
No. of subjects	3334	1906	713	715		
Age, mean (SD)	74.01(6.90)	71.63(5.06)	74.66(6.48)	79.71(8.02)	<.001^a^	<.001^a^
65–74	2150(64.49)	1509(79.17)	422(59.19)	219(30.63)		
75–84	934(28.01)	371(19.46)	245(34.36)	318(44.48)		
85–94	240(7.20)	26(1.36)	45(6.31)	169(23.64)		
95+	10(0.30)	0(0.00)	1(0.14)	9(1.26)		
Gender					<.001^a^	<.001^a^
Female	1815(54.44)	902(47.32)	419(58.77)	494(69.09)		
Male	1519(45.56)	1004(52.68)	294(41.23)	221(30.91)		
Education years					<.001^a^	<.001^a^
0	1024(30.71)	392(20.57)	252(35.34)	380(53.15)		
1–6	1330(39.89)	833(43.70)	288(40.39)	209(29.23)		
> 6	980(29.39)	681(35.73)	173(24.26)	126(17.62)		
Marital status					<.001^a^	<.001^a^
Not married	1014(30.41)	441(23.14)	215(30.15)	358(50.07)		
Married	2320(69.59)	1465(76.86)	498(69.85)	357(49.93)		
Residence					0.109^a^	0.096^a^
Rural	1416(42.47)	812(42.60)	282(39.55)	322(45.03)		
Unban	1918(57.53)	1094(57.40)	431(60.45)	393(54.97)		
Income, mean (SD)	42554.17 (36558.09)	44150.21 (36273.51)	42741.94 (37489.3)	38112.29 (36058.09)	<.001^a^	<.001^a^
Smoking					<.001^a^	<.001^a^
Yes	1057(31.70)	681(35.73)	209(29.31)	167(23.36)		
No	2277(68.30)	1225(64.27)	504(70.69)	548(76.64)		
Drinking					<.001^a^	<.001^a^
Yes	890(26.29)	585(30.69)	184(25.81)	121(16.92)		
No	2444(73.31)	1321(69.31)	529(74.19)	594(83.08)		
Physical exercise					<.001^a^	<.001^a^
Yes	2490(74.69)	1551(81.37)	550(77.14)	389(54.41)		
No	844(25.31)	355(18.63)	163(22.86)	326(45.59)		
DD					<.001^a^	<.001^a^
Better	1635(49.04)	1020(53.52)	323(45.30)	292(40.84)		
Poor	1699(50.96)	886(46.48)	390(54.70)	423(59.16)		
DDS	6.35(1.75)	6.54(1.68)	6.26(1.76)	5.92(1.84)	< 0.01^a^	< 0.001^a^

DD, Dietary Diversity; DDS, Dietary Diversity Score; SD, standard deviation

Data are shown as n (%) for categorical variables, and shown as mean (SD) for continuous variables

^a^ Test with nonparametric tests for income and age, chi-square test for categorical variables and Analysis of Variance (ANOVA) for DDS to compare the characteristics of older adults with different health states

Table 2 shows the percentage of scoring 1 point for different groups, it was more likely for better DD group to score 1 point than the poor DD group, except oil and cereal, other 7 food groups scoring 1 point in better DD group was all higher than that in poor DD group. Except milk and dairy products, other 8 food groups scoring 1 point in better DD group all exceed 70% in 2018 wave. However, fruits in 2011 wave didn’t reach 70% either. In the poor DD group, only cereal, vegetables and oil exceeded 70%.

**Table 2:** Percentage of scoring 1 point for food groups by DD

Food groups	2018		2011	
	Better DD	Poor DD	Better DD	Poor DD
Cereal	99.94%	99.94%	99.91%	99.93%
Vegetables	98.96%	88.38%	98.50%	87.73%
Fruits	73.97%	25.90%	67.17%	20.57%
Soybeans and its products	79.88%	26.56%	84.75%	33.96%
Eggs	95.23%	53.11%	95.60%	48.02%
Meat	95.54%	63.46%	93.92%	59.76%
Fish	80.17%	25.10%	78.58%	23.41%
Milk and dairy products	61.79%	13.68%	52.48%	9.50%
Oil	100.00%	100.00%	100.00%	100.00%

DD, Dietary Diversity

To analyze the effects of DD on health status among older adults, it is necessary to test whether the model is available before fitting the regression model. Test results revealed that p value was less than 0.05, indicating the model with statistical significance and the indicators including DD, age and gender had significant associations with health status, so their internal relationships could be further explored. By the likelihood ratio test, the effects that significantly affected health status, including intercept, DD, age, gender, education years, smoking and drinking etc., which could finally enter into the regression model (Table 3).

**Table 3:** Model fitting information

Model	2018				2011			
	-2 Log Likelihood	Chi-Square	df	p value	-2 Log Likelihood	Chi-Square	df	p value
Intercept Only	6336.147				4835.598			
Final	5284.669	1051.478	26	0.000***	4088.185	747.412	20	0.000***

Tested with chi-square test; df = degree of freedom; * *p* < 0.05; **p < 0.01; ***p < 0.001

Healthy group was the controlled group; multinomial logistic regression was performed to explore determinants for health status. Better DD had a significant association with healthy state (*p* < 0.01). In impaired and dysfunctional groups, poor DD was positively related to impaired and dysfunctional states, indicating older adults with better DD are more likely to be in healthy state. In addition, lifestyles including smoking, drinking and physical exercise were also significantly associated with health status among study participants. Specifically, not smoking were negatively associated with dysfunctional group, and not drinking were negatively related with impaired group in 2018 wave. Not taking moderate physical exercise were positively associated with dysfunctional group in 2018 and 2011 wave. So the consumption of cigarette and alcohol, not taking moderate physical exercise have adverse effects on health (Table 4).

**Table 4:** Multinomial logistic regression results

	2018				2011			
	Impaired group		Dysfunctional group		Impaired group		Dysfunctional group	
	B	P value	B	P value	B	P value	B	P value
Intercept	-8.216	0.000	-15.548	0.000	-7.625	0.000	-14.758	0.000
Age	0.086	0.000	0.170	0.000	0.084	0.000	0.165	0.000
Income	0.000	0.316	0.000	0.132	0.000	0.215	0.000	0.340
Education years (0)	0.520	0.000	0.494	0.002	0.325	0.054	0.739	0.000
Education years (1–6 years)	0.176	0.140	-0.095	0.516	0.099	0.519	0.251	0.200
DD (poor)	0.254	0.010	0.279	0.014	0.395	0.000	0.420	0.001
Gender (female)	0.466	0.000	0.796	0.000	0.372	0.010	0.738	0.000
Marital status (not married)	-0.139	0.205	0.114	0.338	0.136	0.259	0.269	0.036
Physical exercise (no)	0.197	0.078	1.117	0.000	0.125	0.316	1.107	0.000
Smoking (no)	-0.201	0.205	-0.441	0.016	0.000	0.998	-0.036	0.822
Drinking (no)	-0.382	0.010	-0.160	0.374	0.068	0.616	0.125	0.413

DD, Dietary Diversity

Age and gender were also statistical significant (*p* < 0.05) to health status among older adults as shown in Table 4. Health impairment and dysfunction were classified into the unhealthy group, and binary logistic regression was employed to analyze the effects on health status of the elderly by different age and gender groups. Test results revealed that the interaction of age, gender and DD on health status in 2018 wave was statistical significance. DD was more significantly associated with health status of male older adults (*p* < 0.001), with odds ratio (95% CI) of 1.46 (1.13, 1.88) and the younger elderly (*p* < 0.05), with odds ratio (95% CI) of 1.22 (1.04, 1.44) than female older adults (*p* > 0.05), with odds ratio (95% CI) of 1.23 (0.99, 1.54) and the older elderly (*p* > 0.05), with odds ratio (95% CI) of 1.01 (0.37, 2.78). However, for 2011 wave, only the interaction of age and DD on health status was statistical significance. DD was more significantly associated with health status of the younger elderly (*p* < 0.001), with odds ratio (95% CI) of 1.62 (1.26, 2.08) than the older elderly (*p* > 0.05), with odds ratio (95% CI) of 0.08 (0.31, 1.94) (Table 5).

**Table 5:** Logistic regression analysis of the effects of DD on health status by age and gender

2018							
DD		Male older adults				Female older adults	
		Model 1		Model 2		Model 1	Model 2
OR (95% CI) of health status							
Better		1.56 (1.22,2.00)***		1.46 (1.13,1.88)***		1.26 (1.01,1.57)*	1.23 (0.99,1.54)
Poor		reference		reference		reference	reference
		**Younger elderly**				**Older elderly**	
OR (95% CI) of health status							
Better		1.28 (1.09,1.50)***		1.22 (1.04,1.44)*		1.40 (0.57,3.51)	1.01 (0.37,2.78)
Poor		reference		reference		reference	reference
**2011**							
**DD**	**Male older adults**				**Female older adults**		
	**Model 1**		**Model 2**		**Model 1**		**Model 2**
OR (95% CI) of health status							
Better	1.36 (1.05,1.74)*		1.30 (1.00,1.68)*		1.72 (1.30,2.27)***		1.65 (1.25,2.19)***
Poor	reference		reference		reference		reference
	**Younger elderly**				**Older elderly**		
OR (95% CI) of health status							
Better	1.63 (1.34,1.97)***		1.62 (1.26,2.08)***		0.61 (0.22,1.70)		0.08 (0.31,1.94)
Poor	reference		reference		reference		reference

Model 1 adjusted for age, gender, educational years, marital status, income; model 2 additionally adjusted for smoking,drinking.and physical exercise

DD, Dietary Diversity; OR, Odds Ratio; %, Predicted Probability; CI, Confidence Interval

* *p* < 0.05; **p < 0.01; ***p < 0.001

The percentage of scoring 1 point from various food groups by gender and age confirmed the interaction of DD, age and gender on health status. Male group were more likely with better DD than female group. In 2018 wave, except oil and fruits, the percentage of scoring 1 point from other 7 food kinds in male group was all higher than that in female group. There are 5 kinds of food regularly consumed by the male older adults and female older adults including cereal, vegetables, eggs, meat and oil (the percentage of scoring 1 point reached to 70%). Similarly, there was also a significant difference between the younger and the older groups, except oil, milk and dairy products, the percentage of scoring 1 point from other 7 food kinds in the younger group were all higher than that in older group, the foods mainly consumed by the younger group and the older group include cereal, vegetables, eggs, meat and oil, which proved the relationship between gender, age and DD. In 2011 wave, except cereal, milk and dairy products and oil, the percentage of scoring 1 point from other 6 food kinds in the younger group was all higher than that in the older group. There were 4 kinds of food regularly consumed by the younger elderly and the older elderly including cereal, vegetables, meat and oil (the percentage of scoring 1 point reached to 70%) (Table 6).

**Table 6:** Percentage of scoring 1 point for food groups by gender and age

Food groups	2018				2011			
	Male	Female	Younger	Older	Male	Female	Younger	Older
Cereal	99.41%	99.28%	99.93%	99.83%	99.84%	100.00%	99.91%	100.00%
Vegetables	94.72%	92.61%	94.48%	90.96%	92.22%	92.86%	92.73%	90.29%
Fruits	48.15%	50.61%	49.77%	45.02%	41.75%	40.74%	41.86%	34.95%
Soybeans and its products	56.99%	54.58%	53.17%	51.37%	59.12%	53.62%	56.59%	55.83%
Eggs	76.65%	71.70%	74.03%	73.44%	72.96%	64.90%	69.27%	67.96%
Meat	82.85%	76.37%	79.83%	77.46%	79.48%	69.84%	75.09%	73.30%
Fish	55.56%	49.67%	52.88%	48.38%	49.84%	45.77%	48.09%	46.12%
Milk and dairy products	62.93%	38.50%	36.95%	40.33%	28.93%	28.22%	28.27%	32.04%
Oil	100.00%	100.00%	100.00%	100.00%	100.00%	100.00%	100.00%	100.00%

Finally, in order to test the robustness and universalism of model, DD was categorized into three groups, and similar results were obtained.

## Discussion

In this paper, multinomial logistic regression and data of CLHLS 2018 and 2011 wave were used to analyze the effects of DD on health status of the older adults. In conclusion, there is a significant difference among older adults in health status; the elderly with better DD is more likely to be in healthy state. Besides, smoking, drinking and not taking moderate physical exercise do harm to health of older adults, indicating the consumption of cigarette and alcohol, not taking moderate physical exercise have negative effects on health, which may imply a health friendly lifestyle is more beneficial to health.

Another interesting discovery is that the younger group are more active and beneficial than the older group in terms of acquiring relative adequate nutrition that contributes to better health. This effect has several explanations. Firstly, the younger elderly are more active at participating in activities both at home and in society including preparing food and acquiring nutrients compared with the older elderly. Secondly, the younger elderly are better functioning, especially the physiology function of chewing, salivation secretion, ingestion and absorption, which influence the nutrition intake. As digestive system declines with age, it makes the elderly more vulnerable and sensitive to poor DD [33], which may make the effects on health status among the older elderly less obvious.

The contributions of this study come from three aspects. Firstly, this paper goes a step further to probe into the effects of dietary diversity on health status rather than disease, which is from a more active perspective in aging research on health promotion in practice. Secondly, health status assessment is classified into three states, namely healthy, impaired and dysfunctional state by ADL, IADL and MMSE, which makes the health status quantitatively assessed and cover both physical and cognitive aspects, so it is more active and comprehensive and has a significance to health promotion. In addition, the heterogeneity analysis on the relationship between DD and health status by age and gender is conducive to improve health status by different health strategies when facing different groups. Thirdly, health status as a dependent variable in this paper is multinomial and multi-classified, and multinomial logistic regression is used to explore the effects of DD on health status. And as approaches of behaviour intervention, DD together with lifestyles including smoking, drinking and physical activity are discussed, all these make the research on behaviour intervention theory more linking with practice and helpful to health promotion.

The study has limitations as well. Firstly, the reverse causality should be taken into account, for example, preexisting impaired and dysfunctional states may result in a decline of dietary ability to better DD. Secondly, DD was assessed by frequency of food intake, not a standard food frequency questionnaire (FFQ), which may limit generalization of the study at some degree, although previous studies have shown that food variety diversity is an appropriate method for measuring purposes owing to its simplicity [34]. Finally, the problems involved in the data in the CLHLS questionnaire are not enough to conduct further heterogeneity analysis for different regions.

## Conclusions

The effects of dietary diversity on health status among older adults which has a great significance to behavioral interventions in daily life for health promotion, but related researches are inadequate, for most existing literature mainly focused on DD and diseases which is from a negative perspective. From a comprehensive health and positive perspective, this study explored the effects of DD on health status among older adults by using data of CLHLS 2018 and 2011 wave with multinomial logistic regression method. The results show that DD has a significant positive effect on health status among older adults and plays an essential role in health promotion, smoking, drinking and not taking moderate physical exercise can have a negative impact on health according to this population-based study. The younger elderly benefit more obvious compared with the older elderly in terms of acquiring relative adequate nutrition that contributes to better health, inadequate exercise is also unfavourable for health. So diversified and balanced daily dietary should be strengthened for older adults. To ensure the effects of DD on health, we should keep the nutrient digestion and absorption capacity of the venerable age and add nutrients scientifically when necessary.

In addition, nutrition and health literacy improvement, dietary diversity assessment, variety of dietary assistance services for the elder adults in need is essential to balanced nutrition. Meanwhile, lifestyles include smoking and drinking should be reduced as much as possible while appropriate, regular and moderate physical exercise should be encouraged for improving health of older adults, which has an important implication to the public health practice. The effects of DD on health status may reduce as the physical function degenerates as population aging, so it is also essential to ensure continued and equitable access to disease prevention, treatment and rehabilitation during all stages of life to maintain the ability and function of the aging population as long as possible as United Nations suggests to achieve the goal of healthy aging.

## Data Availability

The datasets used during the current study are available in the Peking University Open Research Data (https://opendata.pku.edu.cn/dataset.xhtml?persistentId=doi:10.18170/DVN/WBO7LK&version=2.1）.

## Abbreviations

DDS: Dietary Diversity Score
DD: Dietary Diversity
CLHLS: Chinese Longitudinal Healthy Longevity Survey
ADL: Activities of Daily Living
IADL: Instrument Activities of Daily Living
MMSE: Mini-Mental State Examination
FAO: Food and Agriculture Organization
SD: Standard Deviation
OR: Odds Ratio
CI: Confidence Interval
Df: Degree of freedom
ANOVA: Analysis of Variance
